# Predictive modeling of battery degradation and greenhouse gas emissions from U.S. state-level electric vehicle operation

**DOI:** 10.1038/s41467-018-04826-0

**Published:** 2018-06-21

**Authors:** Fan Yang, Yuanyuan Xie, Yelin Deng, Chris Yuan

**Affiliations:** 1 Department of Mechanical and Aerospace Engineering, Case Western Reserve University, Cleveland, OH 44106 USA; 2 Chemical Science and Engineering, Argonne National Laboratory, Argonne, 60439 IL USA; 3 Department of Mechanical Engineering, University of Wisconsin, Milwaukee, WI 53211 USA

## Abstract

Electric vehicles (EVs) are widely promoted as clean alternatives to conventional vehicles for reducing greenhouse gas (GHG) emissions from ground transportation. However, the battery undergoes a sophisticated degradation process during EV operations and its effects on EV energy consumption and GHG emissions are unknown. Here we show on a typical 24 kWh lithium-manganese-oxide–graphite battery pack that the degradation of EV battery can be mathematically modeled to predict battery life and to study its effects on energy consumption and GHG emissions from EV operations. We found that under US state-level average driving conditions, the battery life is ranging between 5.2 years in Florida and 13.3 years in Alaska under 30% battery degradation limit. The battery degradation will cause a 11.5–16.2% increase in energy consumption and GHG emissions per km driven at 30% capacity loss. This study provides a robust analytical approach and results for supporting policy making in prioritizing EV deployment in the U.S.

## Abstract (web summary)

The effects of battery degradation on the energy consumption and greenhouse gas emissions from electric vehicles are unknown. Here the authors show that the lifetime of a typical battery is between 5.2 and 13.3 years across the U.S., with an 11.5–16.2% increase in energy consumption and CO_2_ emissions.

## Introduction

The fossil fuel combustion in transportation sector generates 25.8% of total greenhouse gases (GHG) emissions in the U.S.^1^ To mitigate the impacts of ground transportation on climate change, the US Environmental Protection Agency along with the US National Highway Traffic Safety Administration has set a regulatory standard to reduce the average GHG emissions of US fleet passenger cars from 139.8 g km^−1^ in 2016 base level to 88.8 g km^−1^ in 2025^2^.

Electric vehicles (EVs) are widely promoted as clean alternatives to conventional vehicles for reducing GHG emissions from ground transportation. The US federal and many state governments are providing a variety of financial and operating incentives including tax credit, fast lane access, emission test exemption, etc., to promote EV adoption^3, 4^. It is expected that EVs will share 24% of US light-vehicle fleet in 2030^5^.

Current EVs are predominantly powered by lithium ion batteries which undergo a complex degradation process during actual EV operation, dictating the energy storage and generating indirect GHG emissions from the consumed electricity. The electricity consumption and associated GHG emissions from EV operations are determined by EV operating conditions and battery charging/discharging processes. In recent years, some research has been conducted on investigating such operating factors as travel demand^6, 7^, electricity mix^8–10^, operating pattern^10, 11^, and ambient temperature^12, 13^ on electricity consumption and GHG emissions from EV operation, while no study has been conducted considering the battery degradation under EV actual driving conditions in the analyses of the electricity consumption and GHG emissions. In current studies on energy and GHG analysis, the EV batteries are simply assumed to have the same lifetime as the vehicles^6–13^, or consider battery replacement at certain cut-off mileage^14, 15^. But in actual EV operation, battery degradation is gradually happening along time under specific driving conditions, and the battery degradation affects the EV electricity consumption and GHG emissions in three ways: decreasing driving range due to reduced capacity, decreasing charging/discharging efficiency due to increasing resistance, requiring battery replacement when the capacity is dropped to the battery degradation limit^16^.

In general, EV battery degradation undergoes two processes: one is the cycling capacity loss due to the internal solid-electrolyte interphase (SEI) layer growth, structure degradation of the electrodes and cyclable lithium loss during the battery charging/discharging process, as mainly dictated by the number of battery charging/discharging cycles; the other is the calendar capacity loss due to battery self-discharge and side reactions during energy storage period, as mainly determined by the state of charge, aging time, and ambient temperature, particularly the high temperatures to which the battery is exposed^16–18^. Due to the largely different operating conditions across the U.S., the EV battery degradation, electricity consumptions, and GHG emissions in each state are largely different.

A predictive analysis of the battery degradation and its effects on energy consumption and GHG emissions from US state-level EV operation is currently unavailable. Here we report a comprehensive and robust analytical approach for quantifying the battery degradation and its effects on energy consumptions and GHG emissions from a mid-size all-battery EV under the average driving conditions in each state of US. From this study, we found that the EV battery degradation is largely different from year to year in each US state. For the annual battery degradation, the calendar capacity loss contributes more to the total capacity loss than the cycling capacity loss. The battery degradation can largely increase the energy consumption and GHG emissions of EV per km driven. These findings from this study can be useful in supporting strategy planning and policy making on sustainable EV deployment across the U.S. in future.

## Results

### Electric vehicle battery degradation under actual operation

The lithium ion battery analyzed in this study is the lithium-manganese oxide (LMO)–graphite battery which is commonly used in EVs, such as Nissan Leaf and Chevrolet Volt. Based on current practice, the average battery cell voltage in this study is set at 3.7 V and each cell operates between 3.4 and 4.1 V. The battery pack consists of 192 battery cells and has an initial 24.15 kWh capacity with 76.7% accessible^19^. A forced convective air cooling condition is simulated (*h* = 25 W m^−2^ K^−1^, fitted from the experimental data of the forced convective air-cooling system^20, 21^) for the battery pack cooling in EV operation. To represent the fresh cell status on a new EV, the initial State of Charge of the battery LMO cathode and graphite anode are set at 0.99 and 0.01, respectively.

Here we developed a comprehensive battery degradation model for the LMO–graphite battery, integrating both the cycling and calendar capacity loss under average driving conditions for a battery EV in each US State for analyses of electricity consumption and GHG emissions (see Methods for details). We developed the cycling capacity loss model based on our previous multi-physics electrochemical model integrating the porous electrode theory, transport phenomena, SEI layer formation, and chemical/electrochemical kinetics^22, 23^. We calculated the calendar capacity loss using a modified Arrhenius-form empirical equation which was established from experimental data correlation and in our analysis is modified to correlate from the hourly timescale^16, 17^. The developed models are validated with actual data reported in literature^24^.

In this study, the battery cycling capacity loss and calendar capacity loss are first calculated separately for the EV under the average driving conditions in each US state, using a monthly–hourly timescale of ambient temperature and separated travel demands for local and highway driving conditions, respectively. The calculated cycling capacity loss and calendar capacity loss are then combined to obtain the annual capacity loss in each state. In the calculation, the driving factors for battery degradation include the annual charging/discharging cycle number which is dependent on the annual travel demand and the driving range of EVs, variations of discharging rates relative to the power outputs required from the battery pack under different driving speeds of EV, as well as the varying temperatures to which the battery is exposed all year round which affects the battery internal kinetics and battery efficiency significantly.

The monthly–hourly travel demands of vehicles in the U.S. are calculated based on the monthly traffic volume data of all registered vehicles in each state, the statistical hourly travel frequency of all surveyed vehicles in a day, and the driving pattern on the percentage of highway vs. local driving in each state (Supplementary Data 3–4). As current EVs are not able to cover the same travel demand as conventional vehicles, in this analysis the travel demand of EVs in each state is proportioned based on the ratio of the vehicle-travel-miles within the driving range of the EVs to the total vehicle-travel-miles. The initial ratio is determined at 71.6% in Alaska to 76.8% in Hawaii, falling between 70.7% and 75.1% in the second year and thereafter corresponding to the battery capacity fading (Supplementary Data 2). The value of the ratio is obtained based on the statistical data compiled by US Federal Highway Administration (FHA)^25^.

**Fig. 1:**
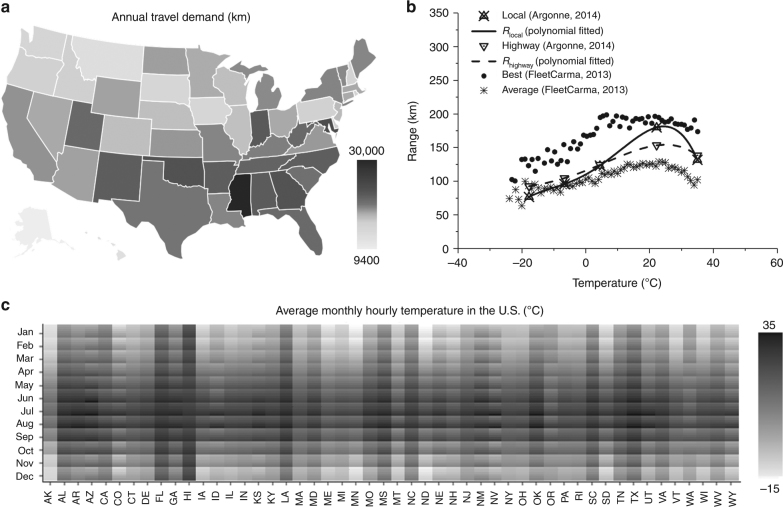
Average state-level operating conditions and initial driving ranges for electric vehicles in the US. **a** Annual travel demand of each state ranging from 9399 km in Alaska to 29,871 km in Mississippi^25^, drawn with Plotly^46^. **b** Initial driving ranges of Nissan Leaf under different ambient temperatures and driving patterns^30, 31^; *R*_local_ and *R*_highway_ are the driving range of electric vehicle under local and highway driving conditions, respectively, the fitted driving range equations are shown in Eqs. (3) and (4) in Methods section. **c** Monthly–hourly average temperature in each US state during 1981–2010 period, ranging between −15 and 35 °C^39^

The driving ranges of a mid-sized EV with a 24 kWh LMO–graphite battery, as reported from 2013 Nissan Leaf on their actual driving in the U.S., are ranging between 64 and 193 km under different driving patterns and temperatures (Fig. 1b), which significantly affect the battery cycling capacity loss and associated GHG emissions during EV driving based on the largely different GHG emission factors (CO_2,eq_ k Wh^−1^) across the U.S. Figure 1 shows the state-level annual travel demand (Fig. 1a), the temperature-dependent driving range of the EV (Fig. 1b), and the monthly–hourly temperature (Fig. 1c) of each US state.

To calculate the cycling capacity loss, the EVs are assumed to cover all those travel demands within the EVs’ actual driving range in each state, based on the statistically compiled vehicle-travel-miles of functional systems in each state^25^. In this analysis, the battery charging/discharging cycle numbers are calculated separately for local and highway driving first, and are then combined based on the driving pattern of the EV in each state of U.S. The cycle numbers are calculated using the proportioned local and highway travel demands of EVs divided by their corresponding driving ranges under the specific ambient temperature in each state.

The EV driving ranges under the monthly–hourly ambient temperatures are modeled and calculated based on the measured data by Argonne National Lab for highway and local driving of 2013 Nissan Leaf with a 24 kWh LMO–graphite battery pack (Fig. 1b)^26^. The EV driving range at 22 °C is 178 and 149 km for local and highway driving, respectively, which can drop by 57% and 40% when ambient temperature goes down to −18 °C, and can drop by 27% and 10% when the temperature goes up to 35 °C^26^. Based on the actual practice, the battery charging current density is set at 0.25 C in this analysis. The real-time discharging rates of the battery pack are determined from the required power outputs of Nissan Leaf, as measured by Argonne National Lab at various driving speeds^26^ and correlated to the typical highway fuel economy test (HWFET) for highway driving and the urban dynamometer driving schedule (UDDS) for local city driving^27^ under different ambient temperatures.

As calculated, the annual cycling capacity losses are between 0.4% in Hawaii and 1.2% in Mississippi in first year, and slightly going up to a range between 0.7% and 1.9% thereafter (Supplementary Data 15). The annual calendar capacity losses for the battery are calculated based on the monthly–hourly temperature in each state and the aging time during the EV battery life, ranging between 4.4% in Alaska and 9.6% in Hawaii in first year, and falling down to a range between 1.0% and 2.2% thereafter (Supplementary Data 16). The cycling capacity loss is dictated by the annual travel demand, with Alaska the smallest at 9399 km and Mississippi the largest at 29,871 km, while the calendar capacity loss is mainly governed by the ambient temperature, with Alaska the lowest average at −2.7 °C and Hawaii the highest average at 24 °C all year round.

**Fig. 2:**
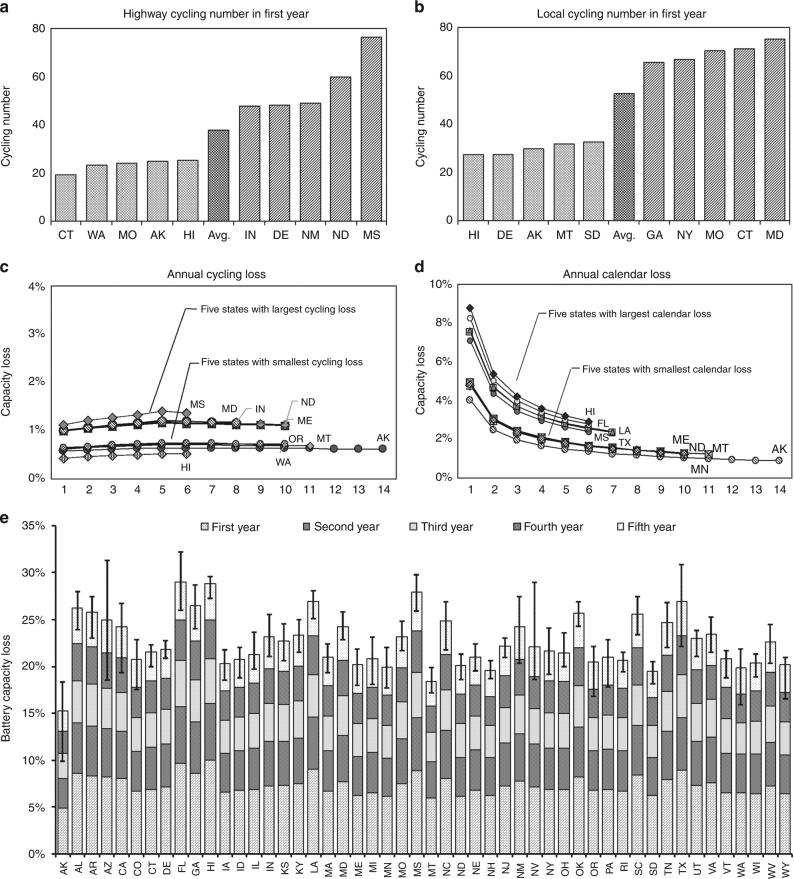
Electric vehicle battery degradation and capacity loss in each state under actual operating conditions. **a** Top five states, bottom five states and the US average of battery cycling number needed for highway driving in first year to meet the annual travel demand. **b** Top five states, bottom five states and the US average of battery cycling number needed for local driving in first year to meet the annual travel demand. **c** Top five and bottom five states for battery annual cycling capacity loss throughout battery life under actual driving conditions, ranging between 0.4% in Hawaii and 1.6% in Mississippi. **d** Top five and bottom five states for battery annual calendar capacity loss during the battery life under each state’s ambient temperature and aging time, ranging between 9.6% in Hawaii and 1% in Alaska. **e** Annual and total battery capacity loss during 5-years operation in each US state under average driving conditions, ranging between 15% in Alaska and 28.7% in Florida. Error bars show the ranges of the battery capacity loss under varying ambient temperatures, travel demands and driving patterns in each state. AL: Alabama, AK: Alaska, AZ: Arizona, AR: Arkansas, CA: California, CO: Colorado, CT: Connecticut, DE: Delaware, FL: Florida, GA: Georgia, HI: Hawaii, ID: Idaho, IL: Illinois, IN: Indiana, IA: Iowa, KS: Kansas, KY: Kentucky, LA: Louisiana, ME: Maine, MD: Maryland, MA: Massachusetts, MI: Michigan, MN: Minnesota, MS: Mississippi, MO: Missouri, MT: Montana, NE: Nebraska, NV: Nevada, NH: New Hampshire, NJ: New Jersey, NM: New Mexico, NY: New York, NC: North Carolina, ND: North Dakota, OH: Ohio, OK: Oklahoma, OR: Oregon, PA: Pennsylvania, RI: Rhode Island, SC: South Carolina, SD: South Dakota, TN: Tennessee, TX: Texas, UT: Utah, VT: Vermont, VA: Virginia, WA: Washington, WV: West Virginia, WI: Wisconsin, WY: Wyoming

The annual battery capacity losses, combining both cycling and calendar capacity loss each year under the actual driving conditions for each state during the first 5 years, are presented in Fig. 2. The battery capacity losses of EVs in each state are different from year to year. The total capacity loss in first year is larger than those in the following years mainly because of the exponential nature of calendar capacity loss resulting from the formation of SEI layer and the reduction of cyclable lithium ion concentrations in the first year of battery operation^16–18^. As calculated, the 1st year total capacity loss of the battery is between 4.9% in Alaska and 10.1% in Hawaii. From the 2nd year, the cycling capacity loss takes an increasing share in the total capacity loss because the calendar capacity loss is decreasing while the cycling capacity loss is relatively stable. As the EV battery degradation limit is currently agreed upon 30%^28, 29^, the EV battery life is calculated ranging between 5.2 years in Florida and 13.3 years in Alaska under current EV driving conditions in each state (Supplementary Figure 3). One battery replacement will be needed for the EV operation in most states, except Alaska and Montana in which a single battery pack can power the EV during the designed 10-year service life.

To validate the developed models and results, the calculated capacity loss values are benchmarked with the measured data on Nissan Leaf for both the calendar capacity loss and total capacity loss, respectively. As shown in Supplementary Table 3, our calculated results for the battery calendar loss after 5 years match the published battery calendar loss very well in Minneapolis, Houston and Phoenix, as reported by National Renewable Energy Laboratory^30^, only with 0.9–1.4% difference. The total capacity loss data as calculated in our study is validated with the actually collected “Plug in America Survey Data” on Nissan Leaf operating under three average high temperatures^24^ (Supplementary Figure 2). The actually reported Nissan Leaf capacity loss data and our calculated total capacity loss values match reasonably well, with the maximum deviations only between 2.9–6.2%, which could be attributed to the differences of battery performance between the averaged and actual operating conditions of the EV, including travel demand, ambient temperature, driving pattern, and travel frequency.

### Energy consumption and GHG emissions

**Fig. 3:**
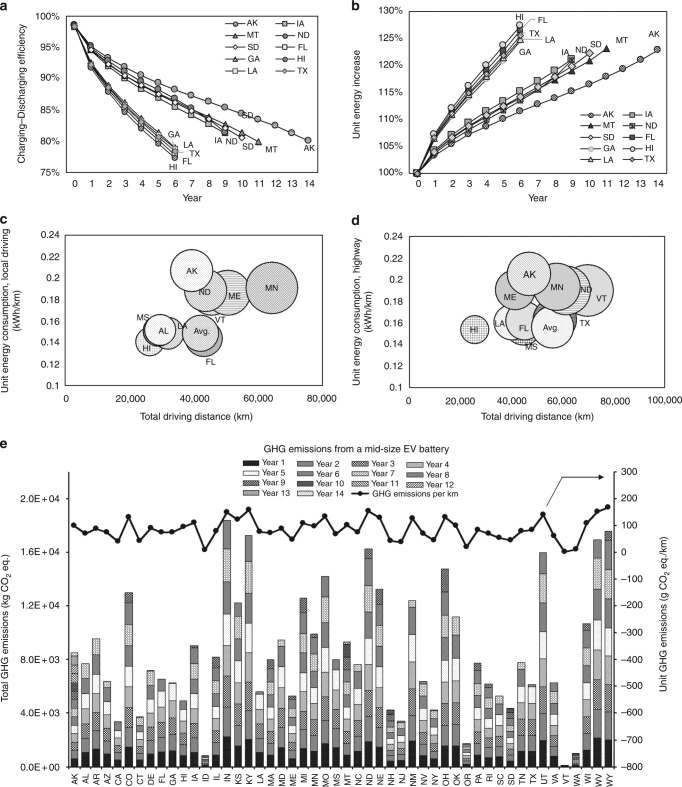
Energy consumption and greenhouse gas emissions from a mid-size electric vehicle battery. **a** Top five and bottom five states on the decreasing rate of battery charging–discharging efficiency, ranging from 98 to 77% during the battery life. **b** Top five and bottom five states on the increasing rate of unit energy consumption, ranging from 100 to 127% upon 30% capacity loss. **c** Top five and bottom five states on unit energy consumption of local driving, ranging between 140 kWh km^−1^ in Hawaii and 207 kWh km^−1^ in Alaska. **d** Top five and bottom five states on energy consumption per km highway driving, ranging between 154 kWh km^−1^ in Hawaii and 205 kWh km^−1^ in Alaska. **e** Unit GHG emissions per km driven and annual total GHG emissions from EV operations in each state, with the unit GHG emissions ranging between 0.6 g CO_2,eq_ km^−1^ in Vermont and 167 g CO_2,eq_ km^−1^ in Wyoming, and the annual total GHG emissions ranging between 8.5 kg in Vermont and 2570.9 kg in Indiana. AL: Alabama, AK: Alaska, AZ: Arizona, AR: Arkansas, CA: California, CO: Colorado, CT: Connecticut, DE: Delaware, FL: Florida, GA: Georgia, HI: Hawaii, ID: Idaho, IL: Illinois, IN: Indiana, IA: Iowa, KS: Kansas, KY: Kentucky, LA: Louisiana, ME: Maine, MD: Maryland, MA: Massachusetts, MI: Michigan, MN: Minnesota, MS: Mississippi, MO: Missouri, MT: Montana, NE: Nebraska, NV: Nevada, NH: New Hampshire, NJ: New Jersey, NM: New Mexico, NY: New York, NC: North Carolina, ND: North Dakota, OH: Ohio, OK: Oklahoma, OR: Oregon, PA: Pennsylvania, RI: Rhode Island, SC: South Carolina, SD: South Dakota, TN: Tennessee, TX: Texas, UT: Utah, VT: Vermont, VA: Virginia, WA: Washington, WV: West Virginia, WI: Wisconsin, WY: Wyoming

The battery capacity loss determines battery life and correspondingly affects the energy consumption of battery pack during electric vehicle driving, which dictates the amount of GHG emissions with state-level variations. In this study, the amount of EV energy consumption is calculated as the amount of electricity drawn from the wall charger for powering the EV to meet the annual travel demand under the average driving conditions in each US state based on the driving range model established on the basis of experimental data from Argonne National Lab^26^ and Fleetcarma^31^ (Fig. 1b). The amount of electricity consumption is calculated with Eq. (7), which considers: actual amount of energy stored in the battery after the annual capacity loss, energy loss on the battery resistance during charging and discharging process, and energy loss from the charger & EV Supply Equipment (EVSE). In this analysis, the charger and EVSE efficiency is set at 85.3% based on Argonne testing data^19^. The battery charging and discharging efficiency, resulting from the battery degradation due to the increasing battery resistance^32^, is calculated using the battery resistance models published in ref. ^33^. The calculated initial charging–discharging efficiency of the EV battery is 98% which decreases at different rates annually in different states. The charging–discharging efficiency drops to 80% in Hawaii in 5th year and 79% in Maine in 9th year (Fig. 3a, Supplementary Data 8).

The battery degradation affects the energy consumption and GHG emissions from EV operations significantly. The unit energy consumption is different from state to state because of the different driving conditions, and is increasing from year to year in each state due to the battery degradation (Fig. 3b). In this analysis, the unit energy consumptions of the EV during highway and local driving are separately calculated (Fig. 3c, d), and then combined together based on the driving pattern of the vehicle in each state. As calculated, the initial energy consumption of the EV operation is ranging between 120.3 kWh km^−1^ in Hawaii and 176.5 kWh km^−1^ in Alaska, corresponding to 80.7 and 87.2 g km^−1^ CO_2,eq_ emissions, based on the GHG emission factors determined by the electricity fuel mix and the imports of electricity in each state using the model from ref. ^34^. At 30% capacity loss, the energy consumption will be increased to 150.2 kWh km^−1^ in Hawaii and 214.8 kWh km^−1^ in Alaska, corresponding to 100.8 and 106.2 g km^−1^ CO_2,eq_ emissions. In general, the energy consumption and GHG emissions from EV operations in the U.S. are increasing by 11.5–16.2% at the recommended 30% battery degradation limit (Supplementary Data 18 and 19). If the EV continues to operate after 30% capacity loss, the energy consumptions and GHG emissions will be largely increased, for instance, by 28% in Mississippi after 10 years driving.

To support strategy planning and policy making on sustainable deployment of EVs, the averaged unit GHG emissions over a single battery life (within 30% capacity loss) and the annual total GHG emissions from the EV driven in each US state are provided in Fig. 3e. On average, the unit GHG emissions from the EV range from 0.6 g km^−1^ in Vermont to 167.1 g km^−1^ in Wyoming, while the annual total GHG emissions are between 8.5 kg in Vermont and 2570.9 kg in Indiana.

## Discussion

In this paper, we report a comprehensive analytical approach for determining battery degradation and its effects on energy consumption and GHG emissions from a mid-size battery EV under the average driving conditions in each state of U.S., using a novel battery degradation model validated with measured data on a 24 kWh LMO–graphite battery pack, to support strategy planning and policy making for sustainable EV deployment in the U.S. It is found that the battery life in each state is quite different under current EV driving conditions, ranging from 5.2 years in Florida to 13.3 years in Alaska. The annual battery degradation of EVs is mainly dependent on the annual travel demand and the ambient high temperature the battery is exposed to. In general, those states with a high annual travel demand above 18,000 km and a high ambient temperature above 28 °C in summer have more severe capacity losses. The temperature-induced calendar loss is dominating the battery degradation, particularly in first year.

The battery degradation causes gradual increasing of battery internal resistance and decreasing of battery charging/discharging efficiency, which results in increasing of unit energy consumption and GHG emissions during EV operations. The energy consumption and GHG emissions can be increased by 11.5–16.2% at the recommended 30% degradation limit for battery replacement, and up to 28% after 10 years driving in the U.S. As EVs are widely promoted as clean alternatives to replace conventional vehicles to reduce GHG emissions from ground transportation sector, the increasing of energy consumption and GHG emissions from battery degradation needs to be considered in the strategy planning and policy making on EV incentives and promotions. Those states with large GHG emission reductions should be provided with enhanced incentives for promoting more EV deployment, while those states with small or no GHG emission reductions should be provided with less or no incentives for EV deployment. Besides, the battery degradation will also lead to required battery replacement, which will add 88.9 GJ equivalent of energy and 5760 kg CO_2,eq_ GHG emissions (Supplementary Figures 4, 5) based on a cradle-to-gate analysis of a 24 kWh LMO–graphite battery pack^35–37^.

A sensitivity analysis is performed on the following four factors: travel demand, electricity fuel mix, battery degradation limit for replacement, and battery capacity accessible ratio (Supplementary Figure 6). The sensitivity analysis reveals that unit GHG emissions per battery are insensitive to annual travel demand and battery capacity accessible ratio. The increasing of annual travel demand from 80 to 120% and battery capacity accessible ratio from 60 to 80% cause only 1% fluctuations in all the states. The increase of battery accessible ratio from 60 to 80% can change the GHG emissions from 97 to 103% of the baseline scenario. On the other hand, reducing the electricity GHG emission factors by adopting clean electricity generation technologies could decrease the unit GHG emissions proportionally.

These results provide fundamental insights how the battery degradation affects the energy consumption and GHG emissions from electric vehicles in different states, adding to the regularities that have previously been identified that the energy use and environmental performance of electric vehicles have significantly regional variability. The modeling approach and results in this paper could be applied by the EV battery designers to evaluate and improve the EV battery performance under different operation conditions by optimizing such battery parameters as total battery capacity, accessible ratio of capacity, rate and depth of charge and discharge, etc. Furthermore, the EV battery manufacturers can apply vehicle-specific strategies and technologies to extend the battery life and improve the vehicle performance. For instance, the battery life can be largely extended if a temperature control system can be applied during the non-operating period of EV, particularly where the ambient temperature is above 28 °C, in such states as Hawaii and Florida. Moreover, the study can enhance the technical services of EV battery manufacturers in such aspects as optimizing the scheduling of battery replacement, inventory planning and control of the battery supply, by providing the accurate degradation data from actual EV operations. From policy perspective, this study provides an accurate modeling approach and results on the battery life, energy consumption and GHG emissions from EVs in each state of U.S., which can be directly used in the US national statistics of energy consumption and GHG emissions from transportation sector. These data and results could be used to support policy making in the electric vehicle incentives to reduce the energy consumption and GHG emissions from the transportation sector more efficiently with specific electric vehicle technologies and the varying state-level operation conditions.

It must be noted that this study is limited to an EV with a 24 kWh LMO–graphite battery pack. Different sizes and chemistries of the battery pack may affect the final results of the study, which could be investigated in future using a modified version of this modeling approach. Also, this study is conducted based on the average state-level data of U.S. Although the uncertainty analysis and the sensitivity analysis investigated the viability of this study to some degree, the possible impacts due to extreme conditions could be significant and needs to be investigated in more details in future. Besides, some assumptions made in this study may also affect the final results. In this study, the driving pattern of the EV on the highway and local operations are modeled based on the US EPA’s HWFET and UDDS driving data. As the driving speeds dictate the discharging profile of battery pack during EV operations, a fast-changing driving pattern could induce more degradations in the battery pack and cause more energy consumption and GHG emissions from the EV on a unit driving distance. The GHG emission factor of marginal electricity mix can also vary at different charging time which needs to be taken into accounts in future studies. The advancement of battery and vehicle technologies may affect the results of the study as well. The increasing of energy density and power density of the battery pack will reduce the energy consumption and GHG emissions from the EV on a unit driving distance. A more-efficient electrified powertrain system will also reduce the unit energy consumption and GHG emissions from the EV. However, fast-charging technologies could induce extra degradation in the battery pack which will increase the unit energy consumption and GHG emissions from the EV if being used on a regular basis.

## Methods

### State-level travel demand of EV in the US

The annual travel demand is the mileage traveled per vehicle in a year. To obtain the EV annual travel demand, we proportioned the traditional vehicle annual travel distance and driving patterns in each state to simulate EV driving in the U.S. These data can be referred to the Highway Statistics Series published by FHA^25^ and have been listed in Supplementary Data 1. In this study, our research target is mid-size EVs, thus we introduce an EV travel demand ratio ($n_r$) to cover those travels within the driving range of EV battery pack: $n_r = m_r/M_r$, where $m_r$ stands for the sum of mileages for all trips within the EV driving range (per charge), which is calculated annually by summarizing the covered one-way trips within the actual EV driving range, with battery degradation and ambient temperature effects considered; $M_r$ is the sum of the mileages of all trips traveled by conventional vehicle. It should be noted here that EV travel demand ratio may also be affected by the fast charging capacity of EV batteries, for instance, the Tesla’s super charger technologies, which could extend the actual driving distance of EV during its service life. The travel demand and driving pattern data in the US are the latest National household travel survey (NHTS) data^25, 38^. The calculated detailed $n_r$ of each state is listed in Supplementary Data 2. Based on the state-level temperature variation and driving condition change^3^, the monthly average travel demand of EV,$D_{m,r}$, is calculated by

1 $$D_{m,r} = \frac{{V_{m,r}}}{{n_v}} \times n_r$$

where $V_{m,r}$ is the monthly average travel volume (km), $n_v$ is the number of registered vehicles, and $n_r$ is the EV travel demand ratio, subscript *r* represents driving pattern (local vs. highway), subscript *m* represents month.

### Travel frequency of EVs in the US

The daily travel frequency of vehicle is the data reported by NHTS^38^. We employ the statistical travel time and duration data in the US, and divide them by the total vehicle traveling time to obtain the hourly travel frequency, as illustrated in Supplementary Figure 1. Using this 24 h travel frequency (*f*_*h*_) of US vehicles, the monthly hourly travel demand (*D*_*m,h,r*_, subscript *h* stands for hour) of EVs is obtained by

2 $$D_{m,h,r} = \left[ {\begin{array}{*{20}{c}} {\frac{{n_rV_{1,r}}}{{n_{\mathrm v}}}} \\ \vdots \\ {\frac{{n_rV_{m,r}}}{{n_{\mathrm v}}}} \\ \vdots \\ {\frac{{n_rV_{12,r}}}{{n_{\mathrm v}}}} \end{array}} \right]\left[ {\begin{array}{*{20}{c}} {f_1} & \cdots & {f_h} & \cdots & {f_{24}} \end{array}} \right]$$

The obtained travel demand of EV is provided in Supplementary Data 3 and  4.

### EV driving range and energy consumption in the US

The driving range of EVs in the US is largely dependent on the EV driving conditions. In this study, the actual testing data of Nissan Leaf from Argonne National Lab^26^ (as shown in Fig. 1b) is fitted to calculate the EV driving range on local and highway under various temperatures, which matches well with the actual driving range data of 2013 and 2014 Nissan Leaf models collected by FleetCarma^31^ under best and average conditions.

3 $$\begin{array}{*{20}{c}} {R_{\rm{local}}} & = & { - 1.1826 \times 10^{ - 4} \times T^4 + 3.75428 \times 10^{ - 5} \times T^3} \\ {} & {} & { + 0.0870367 \times T^2 + 2.83858 \times T + 111.542} \end{array}$$

4 $$\begin{array}{*{20}{c}} {R_{\rm{highway}}} & = & { - 1.68942 \times 10^{ - 5} \times T^4 - 4.50513 \times 10^{ - 4} \times T^3} \\ {} & {} & { - 0.0330376 \times T^2 + 1.95879 \times T + 116.135} \end{array}$$

where *R*_local_ and *R*_highway_ are the driving range of the Nissan Leaf under local and highway driving conditions, respectively. *T* is temperature (°C).

The state-level monthly hourly EV charge–discharge cycles (listed in Supplementary Data 5 and 6) then are calculated using the National Oceanic and Atmospheric Administration (NOAA) data on the US monthly hourly local temperature distribution^39^:

5 $$C_{m,h,r} = \frac{{D_{m,h,r}}}{{R_r(T)}},\,T = \left[ {\begin{array}{*{20}{c}} {T_{1,1}} & \cdots & {T_{1,12}} \\ \vdots & {} & \vdots \\ {T_{24,1}} & \cdots & {T_{m,h}} \end{array}} \right]$$

where $R_r(T)$ is the temperature dependent EV driving range, which represents different load conditions needed by EV sub-systems (e.g., HVAC, radio, etc.) and vehicle internal losses (e.g., alteration of battery and transmission efficiency caused by temperature), and $R_r(T) = R_{\rm{local}}(T)\ {\rm{or }}\ R_{\rm{highway}}(T)$ as given in Fig. 1b, $T$ is monthly hourly temperature (°C). The low driving range of the EV under a low temperature is mainly due to the heater use which is improving over time.

The annual EV charge–discharge cycle numbers ($C$) and energy consumption ($E_t$) are calculated by

6 $$C = \mathop {\sum}\nolimits_{m = 1}^{12} {\mathop {\sum}\nolimits_{h = 1}^{24} {\mathop {\sum}\nolimits_r {C_{m,h,r}} } } ,r = {\rm{local}},\,{\rm{highway}}$$

7 $$E_t = \frac{{C \times E_c}}{{\zeta \times \beta }}$$

where $E_c$ is the energy consumption per charge^19^ (kWh), $\zeta$ is the charger and EVSE efficiency^19^, and $\beta$ is the battery charging-discharging efficiency due to the increase of battery resistance^32, 33^ as calculated by

8 $${\mathrm{\beta = }}\left( {\frac{3}{{\mathrm{2}}} - \frac{{\mathrm{1}}}{{\mathrm{2}}}{\mathrm{ }}\sqrt {1 + \frac{{{{4R}}_{{\mathrm{in}},c}P}}{{\varphi _{\rm{ocv}}^2}}} } \right) \times \left( {\frac{{\mathrm{1}}}{{\mathrm{2}}}{\mathrm{ + }}\frac{{\mathrm{1}}}{{\mathrm{2}}}{\mathrm{ }}\sqrt {1 - \frac{{{{4R}}_{{\mathrm{in}},d}P}}{{\varphi _{\rm{ocv}}^2}}} } \right)$$

where $R_{\rm{in}},c$ and $R_{\rm{in}},d$ are the charging and discharging internal resistance, *P* is the battery power, and $\varphi _{\rm{ocv}}$ is the open circuit voltage. All these calculated data are provided and listed in Supplementary information (Supplementary Data 7–9). Supplementary Data 10 lists the annual energy consumption from EVs in each state during the battery life period in each state.

### EV operating temperature in the US

In this study, the state-level ambient temperature is the average monthly hourly temperature data from the NOAA’s report which is collected from 1981 to 2010 in the U.S.^39^. The detailed temperature data has been summarized and listed in the supplementary Data 11–14.

### EV battery life model

The lithium ion batteries on board of EVs undergo both cycling capacity loss^40^ and calendar capacity loss^17^. In order to precisely calculate the battery life in each state of the US, a comprehensive battery capacity loss model is developed as shown below:

(1) Cycling capacity loss: Cycling capacity loss takes place during the EV charge–discharge cycles. In a LMO–graphite battery, the cycling capacity loss is mainly induced by SEI film growth, electrolyte decomposition and active materials loss. Based on the battery structure: two working electrodes and a separator layer with electrolyte, a pseudo two-dimensional battery capacity fading model is developed and published in our previous work, in which the charge transport process in the battery is formulated by classical Bulter–Volmer equation^23, 41^

9 $$i_F = i_j^0FS_j\left\{ {\exp (\frac{{z\alpha F}}{{RT}}\eta _j) - \exp ( - \frac{{z\alpha F}}{{RT}}\eta _j)} \right\},\,j = {\rm{neg,pos}}$$

where $i_j^0$ is the exchange current density, $F$ is Faraday’s constant, $S_j$ is the specific active interfacial area, $z$ is the transferred electron number, $\alpha$ is the charge transfer coefficient, $R$ is the ideal gas constant, $T$ is the ambient temperature, $\eta _j$ is the overpotential^42,^ neg,pos represent negative carbon electrode and positive LMO electrode respectively.

Considering the impacts of side reactions, the total current transferred can be expressed by

10 $$i_j^{\rm{tot}} = i_F + i_j^s,\,j = {\rm{neg}},\,{\rm {pos}}$$

where $i_j^s$ is the side reaction current, $i_j^s = - i_s^0\exp (z\alpha F\eta _s/RT)$. Meanwhile, the resistance rising induced by excessive SEI growth in negative carbon electrode can be formulated by^23^

11 $$R_f = R_{f,{\rm{ini}}} + R_f(t),\,R_f(t) = L(t){\mathrm{/}}\kappa _p,\,\frac{{\partial L(t)}}{{\partial t}} = - \frac{{i_{\rm{neg}}^sM_p}}{{S_{\rm{neg}}\rho _pF}}$$

where $R_f$ is the total SEI film resistance, $t$ is the time, $R_{f,{\rm{ini}}}$ is the initially formed SEI layer resistance, $R_f(t)$ is the produced film resistance in the cycling, $L(t)$ is the SEI film thickness, $\kappa _p$ is the film conductivity, and $M_p$ and $\rho _p$ are the SEI molecular weight and the density respectively. In the positive electrode, side reactions can also result in severe active materials loss, where the volume fraction change of solid phase (LMO) and the specific area variation can be formulated by^42^

12 $$\frac{{\partial \phi _{\rm{pos}}}}{{\partial t}} = - r_{\mathrm e}S_{\rm{pos}}V_0,\,S_{\rm{pos}} = \frac{{3\phi _{\rm{pos}}}}{{R_{p,\rm{pos}}}}$$

where $\phi _{\rm{pos}}$ is the volume fraction of active LMO, $S_{\rm{pos}}$ is the specific area in positive electrode, $V_0$ is the molar volume of LMO, $R_{p,\rm{pos}}$ is the radius of the spherical electrode particles, and $r_{\mathrm e}$ is the kinetic rate battery electrolyte decomposition, $r_{\mathrm e} = k_{\mathrm e}c_{\rm{H}_{2}O}^2c_{\rm{Li}^ + }$^43^.

In addition to these side reactions, the LMO/carbon battery also includes complicated transport processes. According to ref. ^44^, the charge transport, mass transfer, and energy transport processes in the battery can be formulated by

13 $$\begin{array}{*{20}{c}} {\rm{Charge}} & : & {\nabla \bullet \left( { - \sigma _j^{\rm{eff}}\nabla \phi _{1,j}} \right) = i_j^{\rm{tot}}{\mathrm{,}}} \\ {} & {} & {\nabla \bullet \left( { - \kappa _j^{\rm{eff}}\nabla \phi _{2,j}} \right) + \frac{{2RT\left( {1 - t_ + ^0} \right)}}{F}\nabla \left( { - \kappa _j^{\rm{eff}}\nabla (\ln c_j)} \right) = i_j^{\rm{tot}}} \end{array}$$

14 $$\begin{array}{*{20}{c}} {\rm{Mass}} & : & {\frac{{\partial c_j^{}}}{{\partial t}} = D_j^s\,\frac{1}{{r^2}}\,\frac{\partial }{{\partial r}}\left( {r^2\frac{{\partial c_j^{}}}{{\partial r}}} \right){\mathrm{,}}} \\ {} & {} & {\varepsilon _j\,\frac{{\partial c_j}}{{\partial t}} = \frac{\partial }{{\partial x}}\left( {D_j^{\rm{eff}}\frac{{\partial c_j}}{{\partial x}}} \right) + \frac{{\left( {1 - t_ + ^0} \right)i_j^{\rm{tot}}}}{F}} \end{array}$$

15 $${\rm{Energy}}:\rho c_p\,\frac{{\partial T}}{{\partial t}} + \nabla \bullet \left( { - \lambda \nabla T} \right) = Q_i,\,Q_i = Q_{\rm{rxn}} + Q_{\rm{rev}} + Q_{\rm{ohm}}$$

where $\sigma _j^{\rm{eff}}$ and$\kappa _j^{\rm{eff}}$ are the effective conductivities in solid phase and liquid phase respectively, $\phi _{1,j}$, $\phi _{2,j}$ are the electrode and electrolyte potentials respectively, $t_ + ^0$is the transference number of lithium-ion, $c_j^{}$ is the lithium-ion concentration, $D_j^s$ is its diffusion coefficient in solid materials, $\varepsilon _j$ is the electrode porosity, $D_j^{\rm{eff}}$ is the effective diffusion coefficient^23^, $c_p$ is the specific heat capacity, $\lambda$ is the heat conductivity, $Q_i$ is the heat source term^43^, which is composed of total reaction heat generation $Q_{\rm{rxn}}$, total reversible heat production $Q_{\rm{rev}}$, and total Ohmic heat production $Q_{\rm{ohm}}$. The supplementary formula and expressions can be referred to our previous study on LMO–graphite battery^23^.

The developed mathematical models are solved using finite element package COMSOL Multiphysics and MATLAB software. Two model geometries are applied: a one-dimensional lithium ion battery model and a two-dimensional electrode solid phase model. Two sub-models are coherently coupled in such that the concentration of lithium ions obtained in the 2D solid phase model is projected to the 1D battery model, while the mass flux from 1D battery model is extracted to the 2D solid phase model boundaries. The applied boundary conditions and the associated model parameters are summarized in Supplementary Table 1 and Table 2.

The cycling capacity loss (${\rm{CL}}_{a,{\rm{cyc}}}$) then can be calculated by

16 $${\rm{CL}}_{a,{\rm{cyc}}} = \frac{{\mathop {\sum}\nolimits_{m = 1}^C {I(t_m - t_{m + 1})} }}{{I \times t_1}}$$

where $C$ is the needed charge–discharge cycle number of EV battery in one year to meet the travel demand, $I$ is the average charging current density, and $t_m$ is the time needed to get the EV battery fully charged in $m{\rm{th}}$ cycle.

(2) Calendar capacity loss: The calendar capacity loss takes place during battery energy storage, and mainly caused by battery self-discharge and side reactions. According to ref. ^17^, the battery calendar capacity loss follows Arrhenius-form kinetics, and an empirical expression based on the experimental data is formulated as

17 $${\rm{Cl}}_{a,{\rm{cal}}} = 14,876 \times {\rm{exp}}\left( {\frac{{ - E_a}}{{RT}}} \right)\psi _d\left( {t_h} \right)^{0.5}$$

where ${\rm{Cl}}_{a,{\rm{cal}}}$ is the percentage of calendar capacity loss, $E_a$ is the activation energy, $E_a = 24.5 {\rm{kJ}}$, $R$ is the gas constant, *Ψ*_*d*_*(x)* is the time adjustment function, $t_h$ stands for hour. The Supplementary Note 1, Supplementary Table 3–4, and Supplementary Figure 2 provide a detailed validation of the above battery capacity loss models.

### EV GHG emission

The EV GHG emission from vehicle operation is calculated based on the energy consumptions of EV operation as described in Eq. (7) above and the electricity GHG emission factor in each state of US, as expressed below:

18 $$\begin{array}{*{20}{c}} {\left[ {\rm{GHG}}_{{t,1}} \right.} & \cdots & {{\rm{GHG}}_{t,s}} & \cdots & {\left. {{\rm{GHG}}_{t,50}} \right]} & = & {\left[ {E_{t,1}} \right.} & \cdots & {E_{t,s}} & \cdots & {\left. {E_{t,50}} \right]} \\ {} & {} & {} & {} & {} & {} & { \circ \left[ {U_{G,1}} \right.} & \cdots & {U_{G,s}} & \cdots & {\left. {U_{G,50}} \right]} \end{array}$$

where GHG_*t*,*s*_ is the GHG emissions from vehicle transport energy consumption,  $E_{t,s}$ is the transport energy consumption (kWh), subscript *s* stands for state, $U_{G,s}$ is the unit electricity GHG emission factor (CO_2,eq_ g km^−1^), which is determined from the electricity fuel mix data from the eGRID2012 report published in 2015^34^ and the imports of electricity from other states^45^, as provided in Supplementary Data 17.

### Sensitivity analysis

A sensitivity analysis is conducted to evaluate the viability and robustness of the results relative to the change of the important factors including EV travel demand, electricity GHG emission factor, battery degradation limit for replacement, and capacity accessible ratio. The baseline scenario is with all the current data and results as reported in the paper. The evaluated factors are changed within a reasonable range of their baseline value, and the corresponding changes of the unit GHG emissions (CO_2,eq_ g km^−1^) to the change of each factor is quantified and benchmarked with the baseline scenario, as shown in Supplementary Figure 6a–d and Supplementary Note 2.

### Data availability

All data generated or analyzed during this study are included in this published article as Supplementary Data.

## Electronic supplementary material

Supplementary Information

Supplementary Data 1

Supplementary Data 2

Supplementary Data 3

Supplementary Data 4

Supplementary Data 5

Supplementary Data 6

Supplementary Data 7

Supplementary Data 8

Supplementary Data 9

Supplementary Data 10

Supplementary Data 11

Supplementary Data 12

Supplementary Data 13

Supplementary Data 14

Supplementary Data 15

Supplementary Data 16

Supplementary Data 17

Supplementary Data 18

Supplementary Data 19

Description of Additional Supplementary Files
