# Supplementary material for: Predictive modeling of battery degradation and greenhouse gas emissions from U.S. state-level electric vehicle operation
Source: Nat Commun. 2018 Jun 21;9:2429. doi: 10.1038/s41467-018-04826-0 (PMC6013442; doi:10.1038/s41467-018-04826-0)
Supplement: Supplementary file 1 — Supplementary Information [file 41467_2018_4826_MOESM1_ESM.doc]

Supplementary Information for:

Predictive Modeling of battery degradation and greenhouse gas emissions from U.S. state-level electric vehicle operations

Fan Yang^1,†^, Yuanyuan Xie^2,†^, Yelin Deng^3^, Chris Yuan^1,*^

^1^ Department of Mechanical and Aerospace Engineering, Case Western Reserve University, Cleveland, OH 44106, USA

^2^ Chemical Science and Engineering, Argonne National Laboratory, Argonne, 60439, USA.

^3^ Department of Mechanical Engineering, University of Wisconsin, Milwaukee, WI 53211

*Corresponding author. Tel: +1 216 368 5191; Fax: +1 216 368 6445; E-mail: [chris.yuan@case.edu](mailto:chris.yuan@case.edu)

^†^ These authors contributed equally to this paper.

Supplementary Figures 1-6

Supplementary Tables 1-4

Supplementary Notes 1-2

Supplementary References


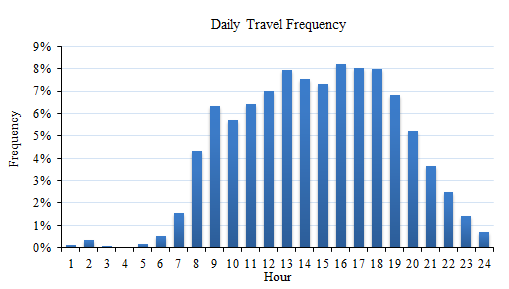


*Supplementary Figure 1. The average travel frequency in the U.S.^1^*


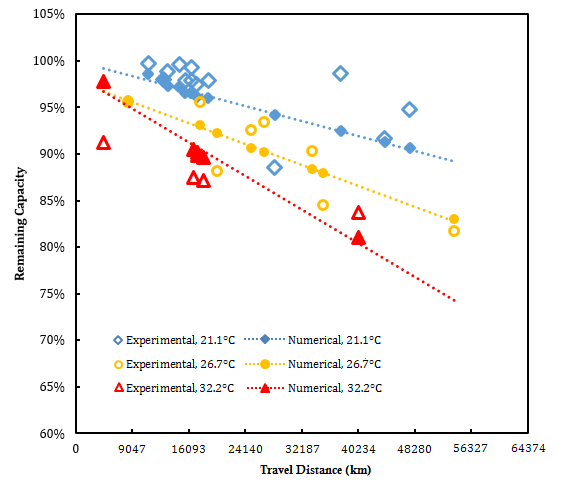


*Supplementary Figure 2. Comparison between our calculated results and actual measured battery capacity loss data^6^*

*
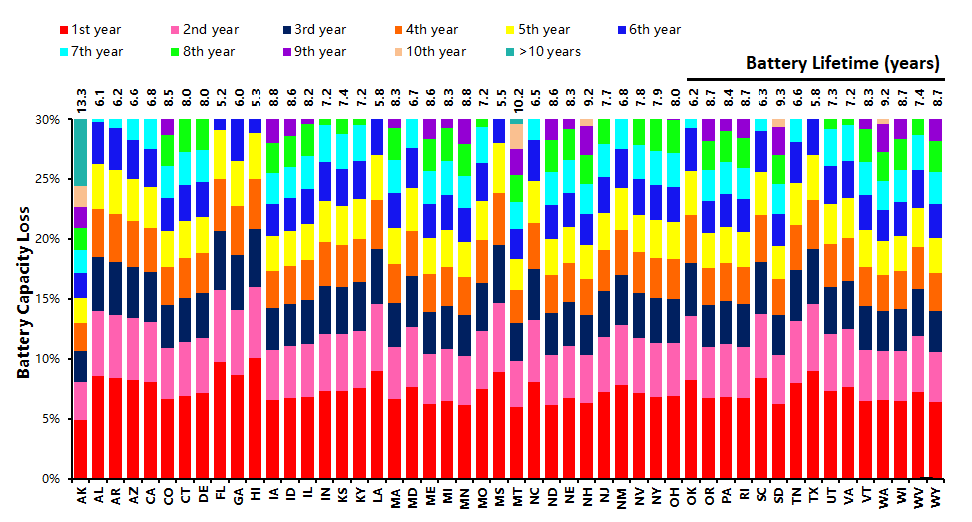
*

*Supplementary Figure 3. The EV Battery lifetime in each state of U.S. with 30% battery degradation limit*

*Supplementary*
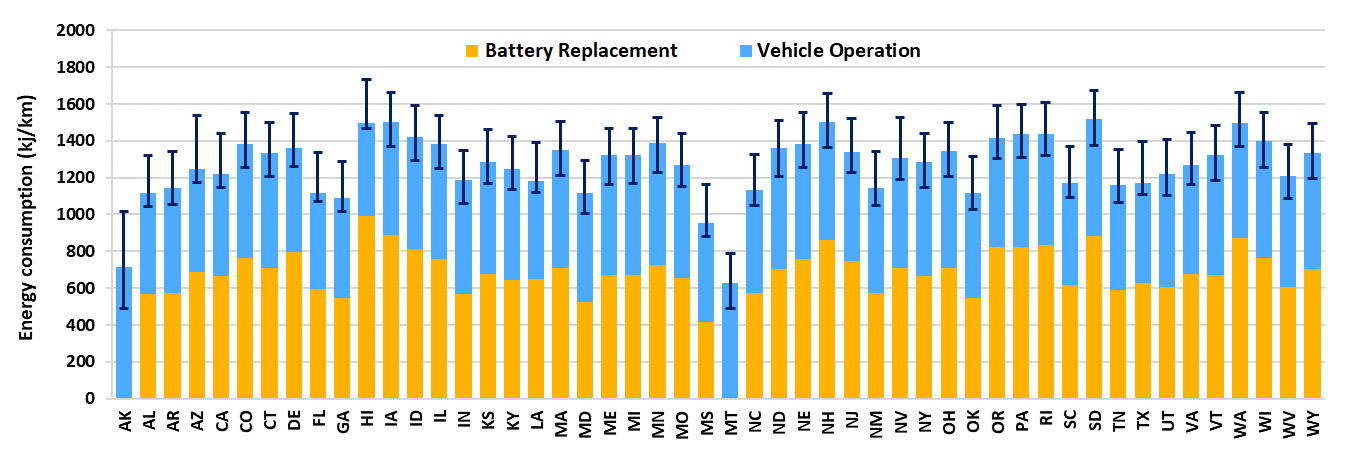
 *Figure 4. Unit energy consumption per km driven from EV operation and battery replacement during a 10-year service life in the U.S. Error bars indicate the uncertainty of unit energy consumption due to the varying operating conditions in each state of U.S.*

*Supplementary*
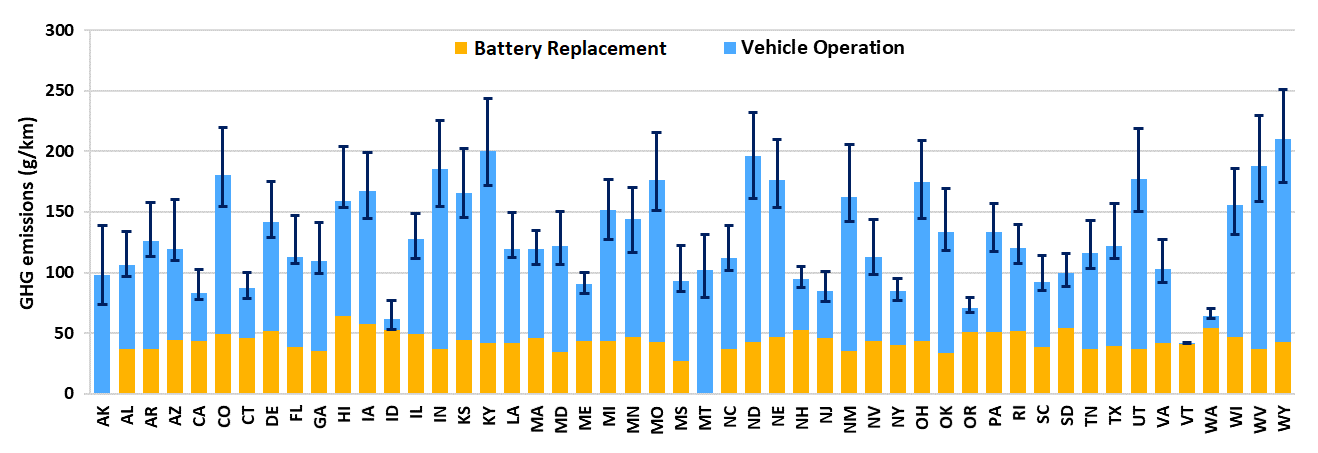
 *Figure 5. Unit GHG emission per km driven from EV operation and battery replacement during a 10-year service life in the U.S. Error bars show the ranges of the battery capacity loss under extreme ambient temperatures, travel demands and driving patterns in each state.*


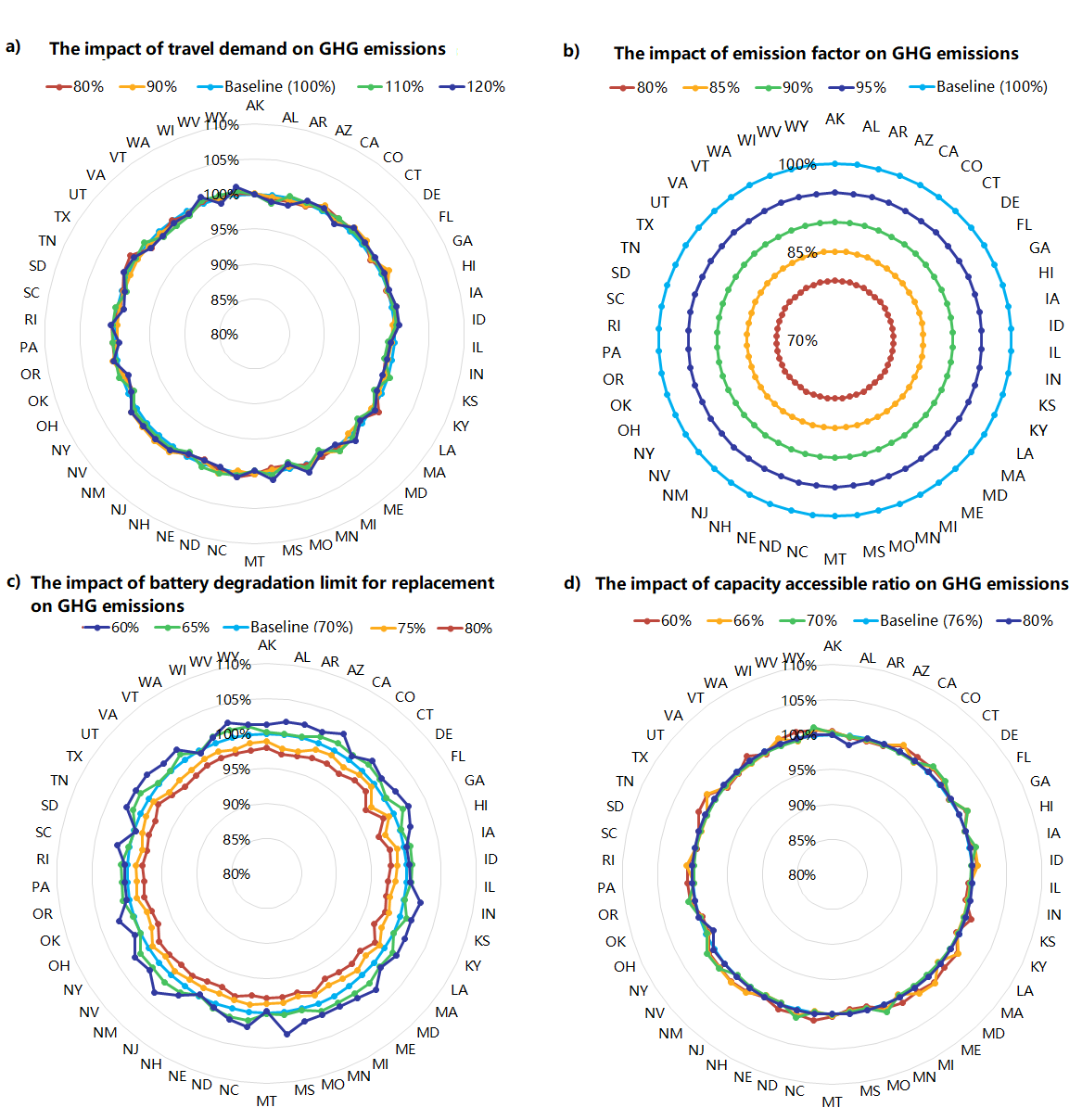


*Supplementary Figure 6. Sensitivity analysis of selected factors on unit GHG emissions per battery. a) the impact of travel deman on GHG emissions b).the impact of emission factor on GHG emissions c).the impact of battery degradation limit for replacement on GHG emissions d). the impact of capacity accessible ratio on GHG emissions*

*Supplementary Table 1 Modeling parameters^2,3^*

| Positive electrode thickness (Cathode): |  | Exchange current of SEI  formation: |  |
| --- | --- | --- | --- |
| Separator thickness: |  | Density of SEI film: |  |
| Negative electrode thickness (Anode): |  | The reference potential  of side reaction, : | $4.2\text{ V}$ |
| Anode particle radius: |  | The activation energy, E_0_: |  |
| Cathode particle radius: |  | Reaction rate constant, k_e_: |  |
| Cationic transport number: | $0.363$ | Bruggeman coefficient: | $1.5$ |
| Li-diffusivity in anode  solid phase: |  | Exchange current density in cathode, : |  |
| Li-diffusivity in cathode  solid phase: |  | Anode solid phase conductivity: |  |
| Exchange current density in anode, : |  | Cathode solid phase conductivity: |  |
| Volume fraction of anode  solid phase: | $0.3$ | Initial electrolyte salt concentration: |  |
| Volume fraction of cathode solid phase: | $0.47$ | Electrolyte salt diffusivity: |  |
| Maximum solid phase  concentration: |  |  |  |

*Supplementary Table 2 Boundary conditions*

| Interface |  |  |  |  |
| --- | --- | --- | --- | --- |
| Charge:  Mass:  Energy: | Applied Current  Insulation  Convective heat transfer | Continuity  Continuity  Continuity | Continuity  Continuity  Continuity | Potential zero  Insulation  Convective heat transfer |
| Particle | $r=0:Zero\text{ }flux$ | | $r=R:Mass\text{ }flux$ | |

*Supplementary Table 3. Calendar loss of Leaf battery after 5 years’ operation^4^*

| City | Our calculation | Experimental data | errors |
| --- | --- | --- | --- |
| Phoenix, AZ | $26.58\%$ | $27.91\%$ | $\leq1.4\%$ |
| Houston, TX | $21.77\%$ | $22.66\%$ | $\leq0.9\%$ |
| Minneapolis, MN | $15.67\%$ | $14.46\%$ | $\leq1.2\%$ |

*Supplementary Table 4. Comparison of numerical and actual total capacity loss of Nissan Leaf battery at 21.1ºC, 26.7ºC, 32.2ºC*

| 21.1ºC validation on reported and calculated remaining capacity | | | |
| --- | --- | --- | --- |
| Mileage | Reported data^6^ | Our calculated data | Deviation |
| 10356 | 99.71% | 98.62% | 1.09% |
| 12429 | 98.04% | 97.91% | 0.13% |
| 13115 | 98.84% | 97.28% | 1.56% |
| 14764 | 99.64% | 97.18% | 2.46% |
| 16419 | 99.24% | 96.54% | 2.70% |
| 15598 | 97.89% | 96.59% | 1.30% |
| 16425 | 97.89% | 96.54% | 1.35% |
| 17252 | 97.41% | 96.06% | 1.35% |
| 18903 | 97.89% | 95.96% | 1.93% |
| 28307 | 88.52% | 94.19% | -5.67% |
| 43998 | 91.71% | 91.29% | 0.42% |
| 47567 | 94.81% | 90.63% | 4.18% |
| 37773 | 98.62% | 92.44% | 6.18% |
| 26.7ºC validation on reported and calculated remaining capacity | | | |
| Mileage | Reported data^6^ | Our calculated data | Deviation |
| 17811 | 95.51% | 93.11% | 2.40% |
| 24985 | 92.49% | 90.63% | 1.86% |
| 26911 | 93.37% | 90.12% | 3.25% |
| 20181 | 88.11% | 92.19% | -4.08% |
| 33672 | 90.27% | 88.32% | 1.95% |
| 35348 | 84.47% | 87.88% | -3.41% |
| 53955 | 81.70% | 82.94% | -1.24% |
| 7480 | 95.58% | 95.73% | -0.15% |
|  | | | |
| 32.2ºC validation on reported and calculated remaining capacity | | | |
| Mileage | Reported data^6^ | Our calculated data | Deviation |
| 17283 | 89.86% | 90.13% | 0.27% |
| 16740 | 87.47% | 90.44% | 2.96% |
| 18256 | 87.16% | 89.67% | 2.51% |
| 40309 | 83.76% | 81.04% | -2.71% |

Supplementary Note 1

Supplementary Table 3 shows the comparison of calendar loss between our calculated results and NREL’s battery data^4^ in Phoenix, Houston and Minneapolis after 5 years. Currently the 24 kWh LMO-graphite battery on Nissan Leaf is warranted for 30% capacity loss for 5 years or 96,561 km ^5^.

The developed capacity loss model is validated using the reported ‘*Plug In America’s LEAF Battery Survey*’ data^6^. During the charge-discharge cycles, it can be seen from Supplementary Figure 6 that our numerical modeling results on battery remaining capacity match reasonably well with the actually collected experimental data of Nissan Leaf battery under three ambient temperatures at 21.1ºC, 26.7ºC and 32.2ºC.

Supplementary Note 2

Supplementary Figure 6 shows the sensitivity analysis results. Supplementary Figure 6a shows the influence of travel demand on the unit EV GHG emission production, where 80%, 90%, 110% and 120% of the baseline travel demand are investigated and benchmarked. It can be seen that the change of travel demand slightly affects the unit GHG emissions for around 1%. This result can be easily explained by the fact that by changing the travel demand, the battery cycling loss status changes correspondingly. However, the subsequent impacts are minimal on the unit GHG emissions due to the tradeoff between the change of travel distance and battery efficiency.

The electricity GHG emission factor reflects local fuel mix for electrical power production. Supplementary Figure 6b studies the impact of local GHG emission factor on the EV GHG emission production. Considering the GHG emission factor is typically decreasing along time due to the introduction of clean energies into the mix, here we defined the new scenarios with 95%, 90%, 85% and 80% of the baseline GHG emission factor. As shown, GHG emissions produced from EV operation can be significantly reduced by decreasing the GHG emission factor, which shows that 80% of the baseline emission factor can lower 20% GHG emissions in all the states. Our sensitivity analysis results clearly show that the GHG emissions from EV operation are linearly affected by the GHG emission factor.

Since the EV battery degradation limit for replacement can affect both the battery life and travel distances, here the battery degradation limit for replacement is investigated as a sensitivity factor, as shown in Supplementary Figure 6c. In comparison with the baseline value at 70% (e.g., Nissan Leaf), four new scenarios: 60%, 65%, 75% and 80%, are defined and evaluated in this sensitivity analysis. It can be seen that the change of the battery degradation limit can generate different impacts, ranging from 96.8% to 103.4%, on the unit EV GHG emissions among U.S. states. The decrease of battery degradation limit for replacement from 70% to 65% or 60% will increase the battery lifetime but decrease the average charging-discharging efficiency and hence increase the unit GHG emissions. However, when the battery degradation limit for replacement increases from 70% to 75% and 80%, the battery will have a shorter battery life, but a higher charging-discharging efficiency and a higher remaining capacity and thus will reduce the average unit GHG emissions.

The battery capacity accessible ratio is another important factor since it determines the accessible amount of energy storage and the operating performance of the battery on the discharge window. In Supplementary Figure 6d, four new battery accessible ratios, 60%, 66% (e.g., Chevrolet Volt), 70% and 80%, are investigated to benchmark with the baseline scenario (76.7%). The results show that the impacts of battery accessible ratio on the unit GHG emissions is insignificant. The increase of battery accessible ratio from 60% to 80% can increase the GHG emissions from 98.6% to 101.4% of the baseline scenario. Similar to travel demand, the battery accessible ratio affects the unit GHG emissions by changing the battery capacity loss speed and subsequently charging-discharging efficiency. The tradeoff between the travel distance and efficiency results in the irregular performance.

**Supplementary References**

1. United States Federal Highway Administration. National household travel survey (NHTS)2009. 2011; Available from: http://purl.access.gpo.gov/GPO/LPS89002.
2. Xie, Y., Li, J., and Yuan, C. Multiphysics modeling of lithium ion battery capacity fading process with solid-electrolyte interphase growth by elementary reaction kinetics. Journal of Power Sources, 2014. 248: p. 172-179.
3. Xie, Y. and C. Yuan. An integrated anode stress model for commercial LixC6-LiyMn2O4 battery during the cycling operation. Journal of Power Sources, 2015. 274: p. 101-113.
4. Smith, K., T. Markel, and A. Pesaran. PHEV battery trade-off study and standby thermal control. in 26th Int. Battery Seminar & Exhibit, Fort Lauderdale, FL. 2009.
5. Nissan Motor Co., Ltd. Lithium-ion battery limited warranty. 2015 [cited 2015 August, 20th]; Available from: <http://www.nissanusa.com/electric-cars/leaf/charging-range/battery/>
6. Saxton, T. Plug In America's LEAF Battery Survey. 2012 [cited 2015 August, 20th]; Available from: http://www.pluginamerica.org/surveys/batteries/leaf/Leaf-Battery-Survey.pdf.
