# Supplementary material for: Predictive modeling of battery degradation and greenhouse gas emissions from U.S. state-level electric vehicle operation
Source: Nat Commun. 2018 Jun 21;9:2429. doi: 10.1038/s41467-018-04826-0 (PMC6013442; doi:10.1038/s41467-018-04826-0)
Supplement: Supplementary file 21 — Description of Additional Supplementary Files [file 41467_2018_4826_MOESM21_ESM.pdf]

Description of Additional Supplementary Files:

Supplementary Data 1. Travel demand and driving patterns in each state of U.S

Supplementary Data 2. Electric vehicle travel ratio in each state of the United States

Supplementary Data 3. Monthly Hourly Average Local Travel Demand in each state of the United States

Supplementary Data 4. Monthly Hourly Average Highway Travel Demand in each state of the United States

Supplementary Data 5. First Year Monthly Hourly Average Local-driving charge-discharge cycles in each state of the United States

Supplementary Data 6. First Year Monthly Hourly Average Highway-driving charge-discharge cycles in each state of the United States

Supplementary Data 7. Increasing battery resistance along time in each state of the United States

Supplementary Data 8. Charging-Discharging efficiency in each state of the United States

Supplementary Data 9. EV energy consumption per charge without battery replacement in each state of the United States (kWh)

Supplementary Data 10. Annual energy consumption from EVs considering travel demand reduction from battery degradation without battery replacement in each state of the United States (kWh)

Supplementary Data 11. Monthly average temperature in each state of the United States (°C)

Supplementary Data 12. 30-year averaged monthly hourly average temperature in each state of the United States

Supplementary Data 13. 30-year averaged monthly hourly highest temperature in each state of the United States

Supplementary Data 14. 30-year averaged monthly hourly lowest temperature in each state of the United States

Supplementary Data 15. Battery cycling capacity loss along time in each state of the United States

Supplementary Data 16. Battery calendar capacity loss along time in each state of the United States

Supplementary Data 17. Electricity GHG emission factor in each state of the United States

Supplementary Data 18. Unit Energy consumption considering battery degradation in each state of the United States (Wh/km)

Supplementary Data 19. Unit GHG emissions considering battery degradation in each state of the United States (g CO_2/km)
